# C. the difference: utilizing daily review and electronic medical record automation to optimize pediatric *Clostridioides difficile* testing

**DOI:** 10.1017/ash.2026.10844

**Published:** 2026-09-09

**Authors:** Dara Grell, Christina Jockel, Zachary Aldewereld, Margot Stein, Jillian Regier, Hannah M. Creager, Terri Stillwell, Michael Green, Lindsay Montoya

**Affiliations:** 1 Quality Services, UPMC Children’s Hospital of Pittsburgh, Pittsburgh, USA; 2 Pediatric Critical Care, UPMC Children’s Hospital of Pittsburgh, Pittsburgh, USA; 3 Divisions of Pediatric Critical Care Medicine and Pediatric Infectious Diseases, University of Pittsburgh, Pittsburgh, USA; 4 Department of Pathology, University of Pittsburgh, Pittsburgh, USA; 5 Division of Pediatric Infectious Diseases, University of Pittsburgh, Pittsburgh, USA

## Abstract

**Objectives:**

To optimize the appropriateness and timing of healthcare associated infection (HAI) time frame *Clostridioides difficile* (CD) testing and reduce misdiagnosed CD HAIs (CDIs) in pediatric patients.

**Methods:**

Two phases of interventions were implemented in a tertiary pediatric acute care hospital. Daily batch testing for CD was introduced in 3/2023 to allow for prereview of CD orders falling in the HAI-time frame. Electronic medical records (EMRs) were reviewed to determine appropriateness using standardized criteria. Discussions were held with providers when testing was deemed inappropriate; testing was canceled if they agreed with reviewer’s feedback. Automated hard stops on CD ordering began in 2/2024 for patients receiving laxatives or enemas within 24 hours prior to test order. EMR pop-up messages prompting consideration of CD testing were initiated May 2024 for patients admitted with diarrhea. Pre and postintervention outcome data were compared.

**Results:**

From 4/1/2023 to 12/31/2025, 476 pending CDI tests were reviewed; 31.5% were deemed inappropriate; 69% of these were canceled. Thirteen percent of 400 completed HAI-time frame orders (including reviewed and unreviewed orders) were positive. Average monthly completed HAI tests and CDI rate decreased postimplementation of daily review protocol (*P* < .0001, and *P* = .153, respectively). Further significant decreases in average monthly completed HAI tests and CDI rate were observed following automated reduction initiatives (both *P* < .0001) compared to preimplementation period. Average standardized infection ratio for CD decreased with each implemented intervention.

**Conclusions:**

Reduction of inappropriate testing and reduction of CDIs following this bundle implementation is evidence for effectiveness of daily review and automated initiatives.

## Introduction

*Clostridioides difficile* (*C. diff*) remains a major cause of healthcare associated infections (HAIs), estimated to cause half a million cases in the Unites States (US) each year.^1^ Cases of HAI due to *C. diff* increase the length of hospital stays and account for at least six billion dollars annually in added healthcare costs in the US.^2^ Accurately determining the burden of *C. diff* disease in pediatric patients is difficult due to the increased likelihood of *C. diff* colonization in young children. The colonization rate of patients less than one year of age is estimated to be around 40%, and over 20% for ages one to two years.^3,4^ Rates taper off with increased ages and reaches 0–15% colonization rate in adults.^5^ Other pediatric groups are also at increased risk of colonization, such as those with inflammatory bowel disease (IBD),^6^ oncologic patients,^7,8^ transplant recipients,^9^ and those with cystic fibrosis.^10^

The Centers for Disease Control and Prevention (CDC) estimates that community acquired cases account for 75% of *C. diff* disease in pediatric patients.^11^ CDC reporting criteria for healthcare associated *C. diff* infection (CDI) includes when polymerase chain reaction (PCR) testing results positive and specimen collection occurs on day three or later of inpatient admission. This reporting criteria makes prompt testing critical to avoid inaccurately reporting community acquired infections as CDI. Due to the increased colonization rate in pediatric patients, it is not just important to have timely assessment, but also to evaluate for the presence of true risk factors and alternative causes of diarrhea to avoid reporting colonized patients as CDI cases, particularly when utilizing an antigen reflex to PCR testing algorithm.

In a tertiary pediatric acute care hospital, an increase of CDI in 2021 and 2022 prompted an assessment that revealed that many of these cases had symptoms (diarrhea) on admission, which self-resolved prior to initiation of treatment and were often associated with laxative use or changes in tube feeds. The purpose of this report is to describe the quality improvement (QI) intervention implemented in response to this.

## Methods

### QI team and PDSA cycles

This is a QI initiative implemented in a tertiary pediatric acute care hospital, beginning in March 2023, with data collected from January 1, 2021 through December 31, 2025. The goal of this effort was to improve diagnostic stewardship and reduce the likelihood of ‘misclassification’ of colonization as CDI. The QI team responsible for identifying and addressing the trend in pediatric cases in this setting was composed of 6 Infection Prevention (IP) team members (4 IPs, 2 IP Medical Directors), 1 microbiology laboratory director, and 1 programmatic nurse specialist from the pediatric intensive care unit. Driven by two Plan-Do-Study-Act (PDSA) cycles, the QI team’s project involved implementation of two phases of *C. diff* interventions, ultimately finalizing our current CDI reduction bundle. Steps necessary for the implementation of this QI initiative were approved by the hospital’s medical executive committee and health system’s QI Committee (UPMC Project IDs 4,400 and 4,677).

PDSA Cycle 1 focused on provider education and implementation of *C. diff* order review. Beginning in early March 2023, education regarding indications for ordering *C. diff* testing was distributed to all clinicians via email. This education was reinforced through monthly CDI Reduction Committee meeting discussions with division leaders and inpatient unit stakeholders. Beginning in mid-March 2023, implementation of *C. diff* order review was accomplished through shifting to once daily batch testing of *C. diff* specimens. This allowed for prereview of pending HAI-time frame *C. diff* test orders Monday through Friday by the IP team. Orders were initially screened to identify patients who had been in the hospital for at least three calendar days using a prompted electronic report. The electronic medical records (EMRs) of identified patients were reviewed using standardized criteria to determine testing appropriateness. If testing was deemed appropriate, no action was taken by the reviewer, and the test was run. If testing was deemed inappropriate, discussions with the clinical teams about order necessity were initiated. *C. diff* orders were canceled if the clinical team agreed with reviewer feedback. If the clinical team disagreed with reviewer feedback, the *C. diff* test was run.

Data was regularly reviewed and discussed during monthly meetings with the QI team and key clinical stakeholders, including clinician leaders from pediatric critical care, oncology, stem cell transplantation, and gastroenterology. Interim review of data revealed that laxative use at the time of HAI-time frame *C. diff* order placement was the most frequent reason that an order was considered ‘inappropriate’. Data review also identified that delays in placing *C. diff* orders, despite presence of relevant symptoms on calendar day 1 of the patient’s admission, resulted in ‘misclassification’ of multiple children as having healthcare associated *C. diff* infections due to date of specimen collection falling within the HAI time frame. PDSA Cycle 2 focused on addressing these two identified gaps in the previous education phase by utilizing EMR automation. Education regarding these automated initiatives was distributed to all hospital clinicians and providers via email from executive leadership in mid-February 2024.

PDSA Cycle 2 began in late February 2024 when the QI team implemented an EMR-driven, automated hard stop on *C. diff* ordering if laxative or enema medications were administered to the patient within 24 hours prior to test order placement. In May 2024, automated pop-up messages prompting providers to consider *C. diff* testing for patients presenting with potentially infectious diarrhea on admission began posting in patient EMRs. During the interim period prior to implementation of automation components, the hospital’s hematology/oncology inpatient unit partnered with the QI Team to trial the on-admission ordering initiative in their patient population, as they frequently presented to the hospital with relevant symptoms. These two automated projects comprised the second phase of the *C. diff* reduction bundle.

### Definitions and metrics

The primary objective of this effort was to reduce the likelihood of ‘misclassification’ of colonization as CDI through diagnostic stewardship of *C. diff* testing. HAI-time frame test orders are defined as any *C. diff* test order placed or collected on calendar day three or later of a patient’s inpatient admission. The EMRs for patients with identified HAI-time frame orders were reviewed using standardized criteria, including: (1) presence of at least one risk factor (recent antibiotic use, recent history of *C. diff* infection, Crohn’s Disease or IBD diagnosis, etc.); and (2) presence of relevant symptoms, (3) in the absence of other explanations for diarrhea (laxative or enema medication use, tube feeding changes, underlying conditions, etc.). An appropriate order is defined as including (1) the presence of at least one *C. diff* risk factor and (2) presence of relevant symptoms, in the absence of other explanations for diarrhea. An inappropriate order is defined as not meeting the standardized criteria for an “appropriate order.” When interpreting *C. diff* testing results, *C. diff* colonization was defined as the presence of toxigenic *C. diff* (as determined by PCR) in absence of toxin (as determined by the TechLab *C. diff* Quik Check Complete assay) and relevant symptoms or alternative explanations for diarrhea. True *C. diff* infection was defined as a positive *C. diff* toxin result in the setting of relevant symptoms without alternative causes for diarrhea. Our primary outcome metrics included rate of testing appropriateness over time, CDI rate over time, and number of *C. diff* tests ordered. Balancing measures associated with this initiative included reviewing results of all HAI-time frame *C. diff* test orders run regardless of appropriateness determination, and assurance to clinical teams that *C. diff* testing could be re-ordered on subsequent days even if it was initially deemed inappropriate. These measures were chosen to assess whether the developed standardized criteria for testing appropriateness could result in delay of care and subsequent harm.

## Statistical methods

Outcome data from each phase of intervention implementation was retrospectively compared with two years of preimplementation data and assessed for statistical significance using paired t-tests and interrupted time series analysis.

### Interrupted time series analysis

We used segmented linear regression to model infection rates over time. This method estimates the baseline trend in infection rates before any intervention, then tests whether each intervention was associated with an immediate change in infection rates (a ‘level change’) or a change in the trend over time (a ‘slope change’), or both.

A key assumption of linear regression is that observations are independent of one another. In time series data, this assumption can be violated when infection rates demonstrate autocorrelation, such as may sometimes be seen with seasonal variation in some infections. Thus, we assessed for autocorrelation using the Durbin-Watson test, which yields values near 2 when no autocorrelation is present. Our Durbin-Watson statistic of 2.10 indicated no significant autocorrelation, supporting the use of standard linear regression without additional corrections.

### Assessing overall impact of interventions

To quantify the overall impact of the interventions, we compared the estimated infection rate at the end of the study period to a ‘counterfactual’ estimate. The counterfactual represents what we would have expected the infection rate to be if preintervention trends had simply continued without any intervention. A statistically significant difference between the observed and counterfactual rates provides evidence that the interventions meaningfully changed the trajectory of infection rates.

We calculated the standard error for this comparison using the Delta method, a statistical technique for quantifying uncertainty in derived estimates.^12,13^ Two-sided p-values and 95% confidence intervals were computed using the *t*-distribution with appropriate degrees of freedom.

Additionally, pairwise comparisons were performed for both the monthly number of tests performed and monthly CDI rate for each phase of the project compared to the preintervention period (for example, monthly number of tests in the daily review phase compared to number of tests in the preintervention phase, etc). Statistical comparison was made using Welch’s t-test (2-sided).

### Software

All analyses were performed in R version 4.5 in RStudio using tidyverse family of packages as well as the car package for autocorrelation diagnostics and the msm package for Delta method calculations.^14–17^

## Results

From April 1, 2023 to December 31, 2025, 476 pending HAI-time frame *C. diff* tests were reviewed. One hundred fifty (31.5%) were deemed inappropriate; of those, 104 (69%) were canceled with permission from the ordering providers after discussion with IP, while 46 (31%) were run based on providers’ preference (Table 1). Of the total 400 HAI-time frame tests that were completed (including both reviewed and unreviewed orders), 52 (13%) resulted positive. Of all tests deemed inappropriate by the IP team, but run per ordering providers’ decisions, none resulted in true infection (per clinical diagnosis), demonstrating the team’s effective method of determining inappropriateness.

**Table 1.** Appropriateness summary of reviewed HAI-time frame tests from daily review initiative implementation, Q2 2023–Q4 2025 Table 1 long description.

Daily review and appropriateness summary	*C. diff* test orders (Q2 2023–Q4 2025)
Total HAI-time frame tests reviewed:	476
* Deemed appropriate*	326
* Deemed inappropriate*	150
Canceled following IP discussion	104
Run despite IP discussion	46

HAI = Healthcare Associated Infection.

*C. diff* = *Clostridioides difficile*.

The monthly average of completed HAI-time frame tests significantly decreased from 20 to 13 tests completed per month (*P* < .0001) and the average monthly CDI rate showed a nonsignificant decrease from 4.69 to 3.48 CDIs per 10,000 patient days (*P* = .153) with implementation of PDSA Cycle 1. Following implementation of PDSA Cycle 2, the monthly average of completed HAI-time frame tests remained relatively steady compared to implementation of PDSA Cycle 1 (13 to 12 tests completed per month; *P* = .589) and significantly decreased compared to the preimplementation period (20 to 12 tests completed per month; *P* < .0001). Also following implementation of PDSA Cycle 2, the average monthly CDI rate decreased compared to implementation of PDSA Cycle 1 (3.48 to 1.81 CDIs per 10,000 patient days; *P* = .045) and the preimplementation period (4.69 to 1.81 CDs per 10,000 patient days; *P* < .0001) (Table 2). The average standardized infection ratio (SIR) for *C. diff* decreased with each implemented phase of intervention (Table 2).

**Table 2.** Comparison of completed HAI-time frame tests and reported CDIs pre and postimplementation daily review and automated initiatives Table 2 long description.

Pre vs postimplementation comparison	PreImplementation (Q1 2021–Q1 2023)	PDSA cycle 1: daily review implementation (Q2 2023—Q1 2024)	*P* value^*^	PDSA cycle 2: automated initiative implementation (Q2 2024—Q4 2025)	*P* value^*^	*P*-value^+^
HAI time frame tests completed:						
Total	533	150		400		
Average completed tests per month [SD]	20 [4.31]	13 [3.03]	<.0001	12 [2.95]	<.0001	.589
Total reported *C. diff* HAIs	99	22		52		
Average monthly CDI rate per 10,000 patient days [SD]	4.69 [2.17]	3.48 [2.42]	.153	1.81 [1.43]	<.0001	.045
Average quarterly standardized infection ratio (SIR)	.980	.664		.338		

* Represents statistical comparison to PreImplementation period.

+ Represents statistical comparison to PDSA Cycle 1 period.

HAI = Healthcare Associated Infection.

CDIs = *Clostridioides difficile* (*C. diff*) HAIs.

Statistical significance calculated using paired *t*-test analysis.

**Figure 1.**
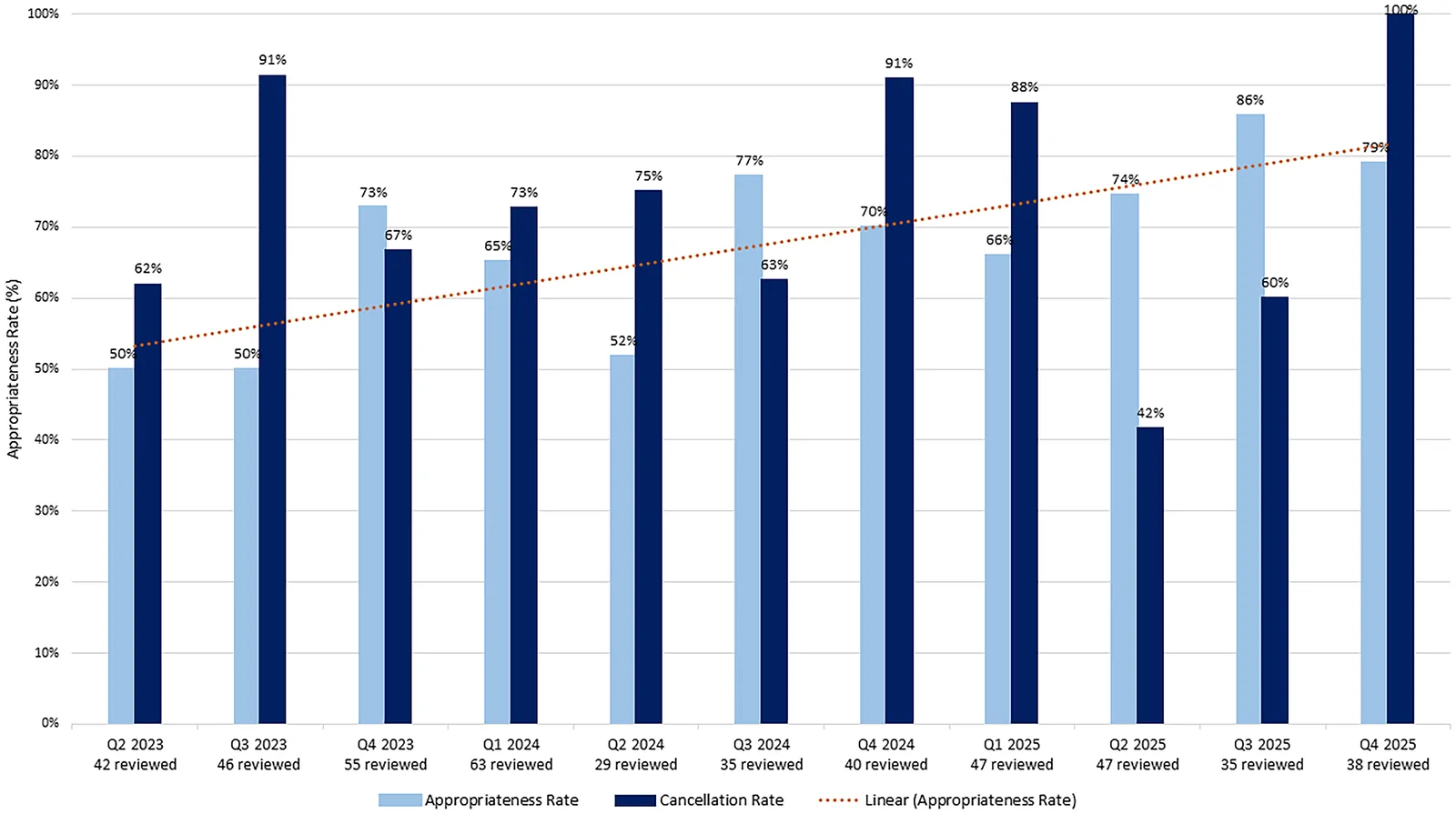
*C. diff* reviews and appropriateness over time (April 2023–December 2025). *Clostridioides difficile* (*C. diff*) testing appropriateness rate (light blue columns), and successful cancellation rate of identified unnecessary tests (navy columns) are plotted by quarter over a two-year period. The linear trend line (orange, dotted) depicts the appropriateness rate over time.

Beginning with the implementation of PDSA Cycle 1, the rate of testing appropriateness increased, and canceled tests decreased over time, suggesting more *C. diff* tests were ordered appropriately (Figure 1). Paired with an increasing cancellation rate over time (Figure 1), the number of completed HAI-time frame tests decreased after daily review implementation, suggesting that inappropriate tests were successfully identified and canceled using the daily review protocol (Table 2).

**Figure 2.**
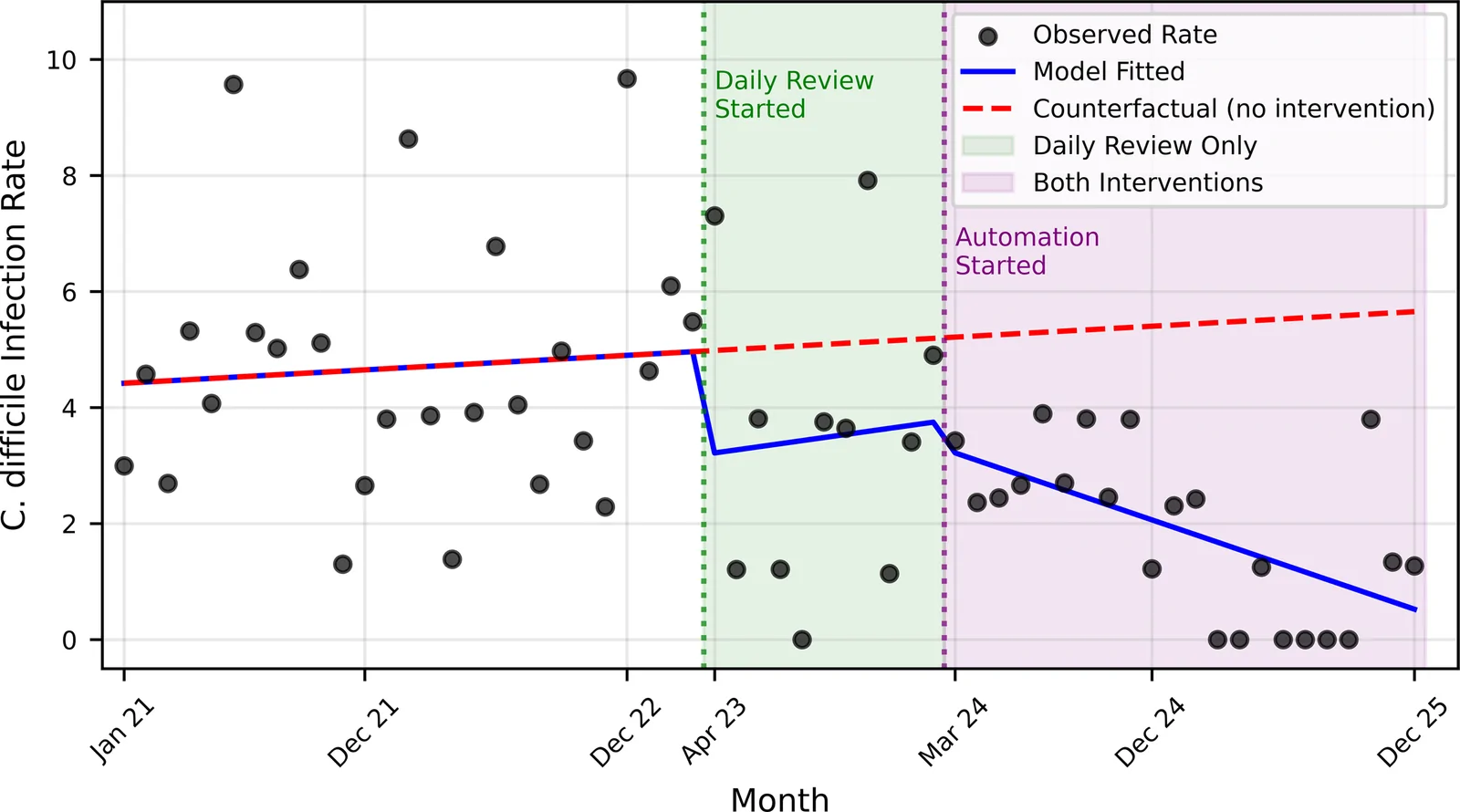
Interrupted time series analysis of *Clostridioides difficile* infection rates over a 60-month period (January 2021– December 2025). Black points represent observed monthly infection rates (number of infections per 10,000 patient days). The blue solid line represents the model-fitted values from the segmented regression model. The red dashed line represents the counterfactual scenario, projecting the preintervention trend forward in the absence of any intervention. Vertical dotted lines indicate the start of indicated interventions. Shaded regions indicate the periods during which each intervention was in effect. At the end of the observation period, the model-estimated rate was 0.52 compared to a counterfactual rate of 5.65, representing a statistically significant reduction of −5.13 (95% CI: −10.02 to −0.24; *P* = .04).

As shown by model-fitted lines in the interrupted time series analysis, the reported CDI rate decreased postimplementation of the daily review protocol and decreased further following implementation of the automated initiatives (Figure 2). The segmented regression model—composed of the baseline time trend and the level and slope changes—explained 34.3% of the variance in *C. difficile* infection rates (adjusted *R* ^2^ = 0.28). In other words, approximately one third of the variation over time can be attributed to the overall trend and intervention effects, with the remaining variation likely attributable to factors not captured by the model, such as random variation and different patient case mixes. Examination of the residuals revealed no substantial autocorrelation, and as previously mentioned, the Durbin-Watson statistic was 2.10, supporting the use of ordinary least squares estimation (Figure 2).

None of the individual intervention coefficients reached statistical significance at the *α* = .05 level. The daily review intervention was associated with a non-significant immediate decrease in infection rate (*β* = −1.80, *P* = .23) and no significant change in trend (*β* = .03 per month, *P* = .87). Similarly, the automation initiative was associated with a non-significant immediate decrease (*β* = −.40, *P* = .78) and a non-significant downward trend change (*β* = −.18 per month, *P* = .37).

Despite the lack of significance for individual coefficients, the cumulative effect of the interventions over time was statistically significant. Using the Delta method to compare the model-predicted rate at the end of the study period to the counterfactual scenario (ie, the projected rate in the absence of interventions), we observed a significant reduction of −5.13 (95% CI: −10.02 to −0.24; *P* = .04). The counterfactual rate at the end of the observation period was 5.65 compared to an observed rate of 1.27, yielding an estimated 77.5% reduction attributable to the combined interventions (Figure 2).

Using the number of canceled orders and 18% CDI percent positivity rate observed in 2022, we estimate avoidance of 16 misdiagnosed CDIs in *C. diff* colonized children since starting these phased interventions.

## Discussion

This implementation demonstrates the cumulative effectiveness of a bundled approach in improving the appropriateness of *C. diff* testing in a pediatric acute care setting, leading to a significant reduction in unnecessary testing and reported CDI cases. Our findings align with the growing recognition that diagnostic stewardship is essential to accurately distinguish between true infections versus colonization (particularly in pediatric populations where colonization rates are notably high^3,4^) and thereby avoid the consequences of inappropriate CDI diagnosis including unnecessary antibiotic exposure, increased healthcare costs, and significant clinical and psychosocial impacts.^2,11^

The team identified 46 test orders that did not align with the testing criteria but were still performed due to clinician preference; of those tests, zero resulted as true infection. This result further validates the IP team’s criteria for test appropriateness and supports following the team’s criteria to safely avoid the potential harm of non-standardized testing, including misdiagnosis, overtreatment, and increased length of hospital stay. Furthermore, the subsequent automated reduction initiatives appeared to sustain and even enhance this downward trend, demonstrating the potential for combined human and automated interventions to optimize diagnostic practices and enhance sustainability of stewardship efforts. The achievement of statistically significant reduction in CDI only after implementation of PDSA Cycle 2 adds to support for the importance of the automated initiatives to the bundle. Such layered approaches can address challenges like provider adherence and variability in clinical judgment, facilitating more reliable implementation of best practices.

Some implementation limitations warrant consideration. The project was conducted at a single tertiary pediatric hospital, which may limit generalizability. Additionally, although the daily review and automated protocols reduced inappropriate testing, the persistence of some inappropriate testing orders highlights ongoing challenges in provider education and engagement. This suggests that stewardship programs must be dynamic and adaptive, incorporating feedback mechanisms and continuing education to foster cultural change among healthcare providers to maximize intended outcomes. Future efforts should explore strategies to integrate stewardship protocols seamlessly into clinical workflows. Lastly, the daily review process required considerable staff time and was conducted only during weekdays, excluding weekends and holidays. This limited surveillance may have allowed some inappropriate tests ordered during non-review periods to go unchecked, potentially impacting overall effectiveness. Future programs should consider resource allocation and explore strategies for extending review coverage or leveraging automation further to address these gaps.

In conclusion, our findings support the implementation of structured diagnostic stewardship programs incorporating both real-time review and automation to reduce inappropriate *C. diff* testing and improve the accuracy of CDI diagnosis in pediatric patients. Such initiatives are essential to mitigate the clinical and economic consequences of misdiagnosis and to promote high-value care in this vulnerable population.

## Table 1. Long description

A table titled ‘Daily review and appropriateness summary’ focuses on C. diff test orders from Q2 2023 to Q4 2025. The table has four rows and three columns. The columns are labeled ’Total HAI-time frame tests reviewed’, ‘Deemed appropriate’, ‘Deemed inappropriate’, ‘Canceled following IP discussion’, and ‘Run despite IP discussion’. The first row shows the total number of tests reviewed: 476. The second row indicates that 326 tests were deemed appropriate. The third row shows that 150 tests were deemed inappropriate. The fourth row shows that 104 tests were canceled following IP discussion. The fifth row shows that 46 tests were run despite IP discussion.

Navigate back to Table 1..

## Table 2. Long description

A table comparing healthcare-associated infection tests and reported Clostridioides difficile infections pre and post implementation of interventions. The table has 4 rows and 7 columns. Column headers are: Pre vs postimplementation comparison, Preimplementation (Q1 2021–Q1 2023), PDSA cycle 1: daily review implementation (Q2 2023—Q1 2024), P value, PDSA cycle 2: automated initiative implementation (Q2 2024—Q4 2025), P value, P value. Row labels are: HAI time frame tests completed: Total, Average completed tests per month [SD], Total reported C. diff HAIs, Average monthly CDI rate per 10,000 patient days [SD], Average quarterly standardized infection ratio (SIR). Row 1 shows the total number of HAI time frame tests completed: 533 pre-implementation, 150 post-implementation of PDSA Cycle 1, and 400 post-implementation of PDSA Cycle 2. Row 2 shows the average completed number of tests per month accompanied by the standard deviation and P value. Pre-implementation was 20 [4.31]. Post-implementation of PDSA Cycle 1 was 13 [3.03] with P value of < .0001 compared to pre-implementation. Post-implementation of PDSA Cycle 2 was 12 [12.95] with P value of < .0001 compared to pre-implementation and P value of .589 compared to PDSA Cycle 1. Row 3 shows the total reported C. diff HAIs: 99 pre-implementation, 22 post-implementation of PDSA Cycle 1, and 52 post-implementation of PDSA Cycle 2. Row 4 shows the average monthly CDI rate per 10,000 patient days, accompanied by the standard deviation and P value. Pre-implementation was 4.69 [2.17]. Post-implementation of PDSA Cycle 1 was 3.48 [2.42] with P value of .153 compared to pre-implementation. Post-implementation of PDSA Cycle 2 was 1.81 [1.43] with P value of < .0001 compared to pre-implementation and P value of .045 compared to PDSA Cycle 1. Row 5 shows the average quarterly standardized infection ratio (SIR): .980 pre-implementation, .664 post-implementation of PDSA Cycle 1, and .338 post-implementation of PDSA Cycle 2.

Navigate back to Table 2..
